# Supplementary material for: Predicting visual function by interpreting a neuronal wiring diagram
Source: Nature. 2024 Oct 2;634(8032):113–23. doi: 10.1038/s41586-024-07953-5 (PMC11446822; doi:10.1038/s41586-024-07953-5)
Supplement: Supplementary file 5 — CRF and ERF predictions for individual Dm3 cells. The CRF is predicted for each target cell by mapping the top five monosynaptic pathways from hexel source types to the target Dm3 type. The ERF is predicted using disynaptic pathways that pass through the top ten intermediary types (ranking from Extended Data Fig. 7). As a set of disynaptic pathways passes from multiple hexel source types through each intermediary to the target, for brevity only one representative starting from a single hexel type is shown, the pathway in the set with maximal strength (red line, Extended Data Fig. 7). The centre of the Tm1–Dm3 CRF (Methods) is indicated by the cyan star, to aid comparison of locations across maps. Next to each map are the maximum and sum over all hexels, in units of 0.01%. In the last panel, all ellipse approximations are shown together for comparison, scaled up by ×3 relative to maps. The dashed ellipse approximates the Tm1–Dm3 CRF, and scale bars indicate one lattice constant and p and q axes. Each file name contains the ID of the target cell in v783. [file 41586_2024_7953_MOESM5_ESM.zip › DataS3/Dm3v/720575940644977175.pdf]

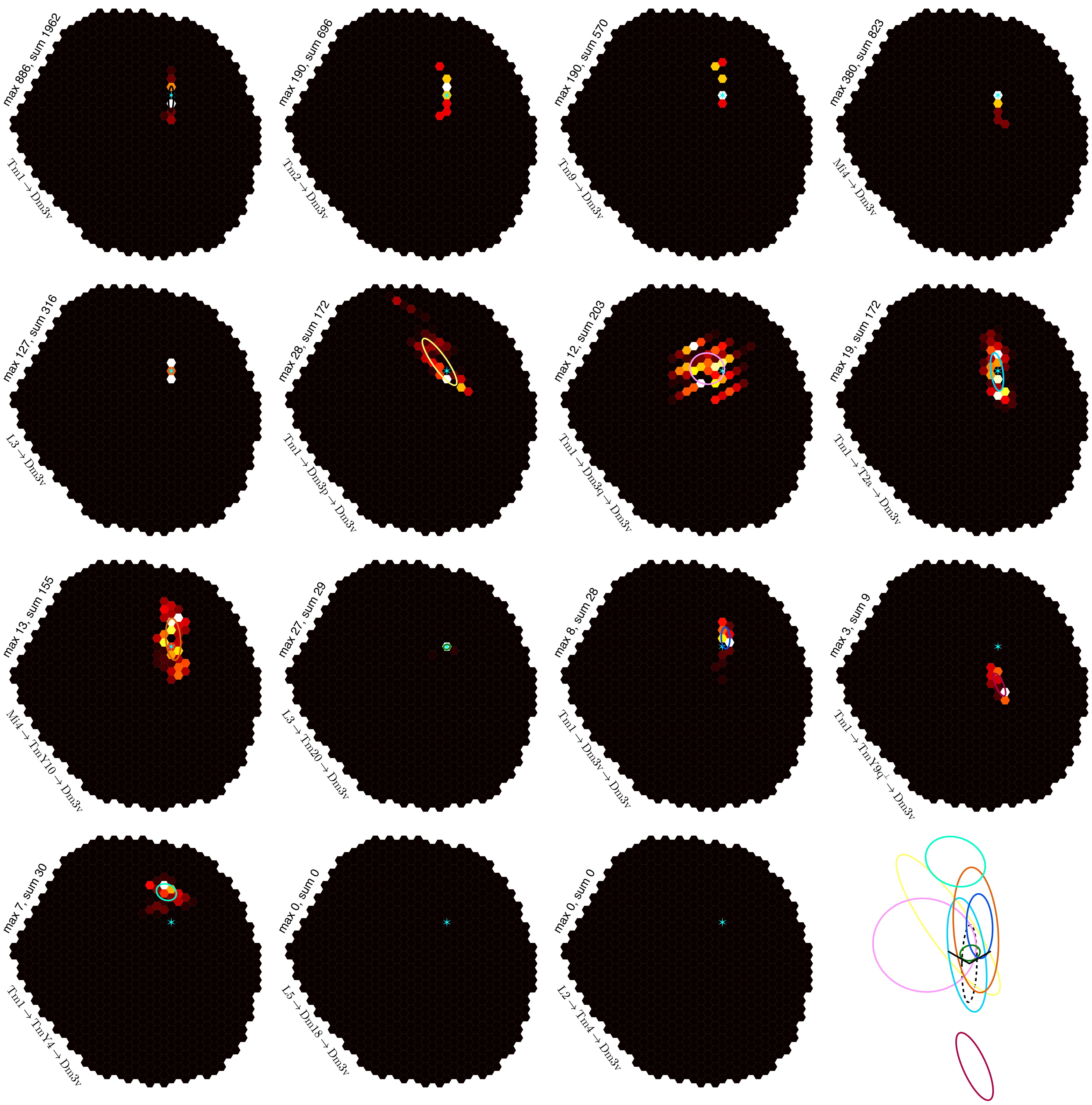
max 886, sum 1962
Tm1 → Dm3v
max 190, sum 696
Tm2 → Dm3v
max 190, sum 570
Tm9 → Dm3v
max 380, sum 823
Mi4 → Dm3v
max 127, sum 316
L3 → Dm3v
max 28, sum 172
Tm1 → Dm3p → Dm3v
max 12, sum 203
Tm1 → Dm3q → Dm3v
max 19, sum 172
Tm1 → T2a → Dm3v
max 13, sum 155
Mi4 → TmY10 → Dm3v
max 27, sum 29
L3 → Tm20 → Dm3v
max 8, sum 28
Tm1 → Dm3v → Dm3v
max 3, sum 9
Tm1 → TmY9q$^{\perp}$ → Dm3v
max 7, sum 30
Tm1 → TmY4 → Dm3v
max 0, sum 0
L5 → Dm18 → Dm3v
max 0, sum 0
L2 → Tm4 → Dm3v
